# Effect of N(Epsilon)-(carboxymethyl)lysine on Laboratory Parameters and Its Association with *β*^S^ Haplotype in Children with Sickle Cell Anemia

**DOI:** 10.1155/2019/1580485

**Published:** 2019-09-15

**Authors:** Uche Samuel Ndidi, Corynne Stephanie Ahouefa Adanho, Rayra Pereira Santiago, Sètondji Cocou Modeste Alexandre Yahouédéhou, Sânzio Silva Santana, Vitor Valério Mafili, Thassila Nogueira Pitanga, Cleverson Alves Fonseca, Junia Raquel Dutra Ferreira, Elisângela Vitoria Adorno, Isa Menezes Lyra, Adekunle D. Adekile, Cynara Gomes Barbosa, Marilda Souza Goncalves

**Affiliations:** ^1^Laboratório de Investigação em Genética e Hematologia Translacional, Instituto Gonçalo Moniz, Salvador, BA, Brazil; ^2^Department of Biochemistry, Ahmadu Bello University, Zaria, Nigeria; ^3^Laboratório de Pesquisa em Anemias, Departamento de Análises Clínicas e Toxicologicas, Faculdade de Farmácia, Universidade Federal da Bahia, Salvador, BA, Brazil; ^4^Fundação de Hematologia e Hemoterapia da Bahia, Salvador, BA, Brazil; ^5^Department of Pediatrics, Faculty of Medicine, Kuwait University, Kuwait

## Abstract

The present study aimed to investigate the association of N^*ε*^-carboxymethyllysine (CML) with laboratory parameters and *β*^S^ haplotypes in pediatric sickle cell anemia (SCA) patients with or without hydroxyurea (HU) therapy. We included 55 children with SCA (SCA_total_), where 27 were on HU treatment (SCA-HU^+^) and 28 without HU treatment (SCA-HU^−^). Laboratory characteristics were determined using electronic methods while CML was measured using competitive ELISA. *β*^S^ haplotypes were determined by RFLP-PCR. Significant increases in MCV and MCH and significant decreases in leukocytes, eosinophils, basophils, atypical lymphocytes, lymphocytes, and monocytes were found in SCA-HU^+^ compared to SCA-HU^−^. SCA-HU^+^ presented significant reduction in aspartate transaminase and lactate dehydrogenase and increase in creatinine levels compared to SCA-HU^−^. CML levels were significantly higher in both SCA-HU^+^ and SCA-HU^−^ compared to the healthy control. In addition, a negative correlation was found between CML and alanine transaminase in SCA-HU^+^ and SCA_total_ (*p* < 0.01). A significant association was found between CML levels and *β*^S^ haplotypes. The results suggest that CML has a role to play in SCA complications, independent of HU therapy.

## 1. Introduction

Sickle cell anemia (SCA) is a monogenic hematological disorder caused by substitution GAG>GTG at the 6^th^ position of the beta globin gene (*HBB*) located in chromosome 11 [1]. SCA patients present a wide variability in clinical manifestations regarding the functions of vital organs as well as frequency and severity of vasoocclusive crises [2, 3]. This can be explained by factors such as fetal hemoglobin (HbF) levels, coexistence of alpha (*α*) thalassemia, haplotypes associated with the *β*^S^ globin gene, oxidative stress, features intrinsic to the red blood cell (RBC), and extracellular environment [4, 5]. Higher HbF levels were associated with improved survival, decreased rates of painful crises, acute chest syndrome, and osteonecrosis [6–9]. Furthermore, Senegal (SEN), Cameroon (CAM), Bantu or Central African Republic (CAR), Benin (BEN), Arab-Indian (ARAB) and more recently the atypical (AT) *β*^S^ haplotype are associated with variable HbF levels and, consequently, variable clinical features [10, 11]. Autooxidation of the HbS along with chronic intravascular hemolysis and ischemia is known to increase the generation of reactive oxygen species (ROS) that potentiates oxidative stress which, in association with other features intrinsic to the erythrocyte and extracellular environment, can mediate vasoocclusion and organ ischemia [5, 12].

Reports showed that advanced glycation end products (AGEs), such as N^*ε*^-carboxymethyllysine (CML), are markers of oxidative stress [13] and have been implicated in normal aging as well as pathophysiology of organ complications in diabetes, atherosclerosis, Alzheimer, and autoimmune inflammatory disease [14–17]. They are a complex group of compounds formed through nonenzymatic reactions between reducing sugars or derivatives (e.g., glucose-6-phosphate) and the N-terminal amino group of arginine and lysine side chains on proteins, lipids, and nucleic acids. It is known that AGEs lead to the formation of covalent cross-links between proteins that may be one of the central underlying processes by which they cause damage [18]. Studies of Nur et al. [15] and Somjee et al. [19] are the only known ones that tried to relate AGEs with sickle cell disease (SCD). They reported increase in AGE levels in SCD patients compared to healthy individuals (HbAA). Their results showed that circulating AGEs may play a significant role in vascular dysfunction, pathophysiology of hemolytic phenotype, and hemolysis-related organ complications such as priapism, leg ulcer, ischemic strokes, and pulmonary hypertension [15, 19].

To improve these clinical features presented by the SCA patients, hydroxyurea (HU) was the only drug approved in 1998 by the Food and Drug Administration (FDA) [20, 21] until 2017 when L-glutamine also became part of this therapeutic arsenal [22]. Reports suggest that HU is a relatively well-tolerated cytotoxic drug with limited side effects in the short term, though there are concerns over its long-term effect on male fertility [23]. The targets and mechanisms by which HU ameliorates clinical complications of SCA remain partially elucidated [24–27]. The efficacy of HU was initially attributed to pharmacological stimulation of HbF, but the fact that clinical benefits occur before its rise suggests that HU could act through other mechanisms [28, 29]. Therefore, understanding the genetic and other factors underlying the variability in the therapeutic effects of HU is critical for prospectively predicting good responders and for designing other effective therapies. The present study was aimed at evaluating the association of CML, the most abundant and researched advanced glycation product [30], with laboratory parameters and *β*^S^ haplotypes in SCA patients with or without HU treatment.

## 2. Materials and Methods

### 2.1. Subjects and Ethical Aspects

The present transversal study carried out between August 2015 and August 2017 was performed with 55 SCA (HbSS) children from the Fundação de Hematologia e Hemoterapia da Bahia (HEMOBA), Salvador, Bahia, Brazil. They were divided into two groups: the SCA-HU^+^ group composed of 27 children with HU therapy (15–25 mg/kg/day) and the SCA-HU^−^ group composed of 28 children without HU therapy. The median ages of the SCA-HU^+^ and SCA-HU^−^ groups were 7 and 6 years, respectively. All individuals were in the steady state of the disease at the moment of enrollment. Steady state is defined as the absence of any acute events and no blood transfusion during the 120 days prior to blood sampling. In addition, 30 healthy age- and sex-matched individuals (HbAA) were recruited from the Clinical Laboratory of the Faculdade de Farmácia at the Universidade Federal da Bahia (UFBA). All procedures were in accordance with the Helsinki declaration and its later amendments. In addition, the study was approved by the Instituto Gonçalo Moniz Ethics research board (1.400.527), and the legal guardians of the children signed the informed consent form before their enrollment in the study.

### 2.2. Laboratory Methods

Hematological analyses were carried out using an automated cell counter, Coulter Count T-890 (Coulter Corporation, FL, USA). The hemoglobin (Hb) profile and HbF levels were investigated by high-performance liquid chromatography (HPLC/VARIANT I; Bio-Rad, CA, USA). Biochemical markers were assessed by immunochemistry assay (A25 system, BioSystems SA, Barcelona, Spain). Serum ferritin was measured by immunoassay using an Access® 2 Immunoassay System X2 (Beckman Coulter, Fullerton, CA, USA). C-reactive protein (CRP), alpha 1-antitrypsin (AAT), and antistreptolysin O (ASO) were measured by immunochemistry (IMMAGE® 800 system, Beckman Coulter, Fullerton, CA, USA). Nitric oxide (NO) was indirectly quantified through nitrite quantification by the colorimetric method at 540 nm [31].

### 2.3. Immunoanalysis of N^*ε*^-Carboxymethyllysine

The detection and quantitative estimation of CML was carried out in the serum of the individuals using an OxiSelect™ CML ELISA Kit according to the manufacturer's instructions (Cell Biolabs Inc., CA, USA). The absorbance was read on a microplate reader (STAT FAX® 2100) at 450 nm, and the CML concentration, in ng/mL, was determined using a CML-BSA standard curve.

### 2.4. Haplotype Analysis

DNA was extracted from leukocytes of individuals following instructions of the commercial DNA isolation kit (QIAGEN, Hilden, Germany), and the analysis of *β*^S^ haplotypes was performed by restriction fragment length polymorphism-polymerase chain reaction (PCR-RFLP) according to the method of Sutton et al. [32].

### 2.5. Statistical Analysis

Statistical analyses were performed using GraphPad Prism 7.0 and SPSS 20.0. *p* values < 0.05 were considered as statistically significant. Quantitative variable distribution was determined by Shapiro-Wilk test. The Mann-Whitney *U* test (nonparametric) and unpaired *t*-test (parametric) were employed for the analysis of two quantitative variables, comparing two groups within the same variable, taking into account the distribution of each variable. Analysis of variance (ANOVA) followed by post hoc multiple range comparison was applied to compare the mean of more than two groups. Spearman's rank correlation coefficient was employed to assess relations between CML and laboratory parameters. For qualitative variables, the Pearson chi-square or Fischer exact tests were performed comparing frequencies between two groups. Results were expressed as the median and 25^th^–75^th^ percentile, number, and percentage, where appropriate.

## 3. Results

### 3.1. Laboratory Markers

Table 1 presents hematological characteristics of SCA-HU^+^ and SCA-HU^−^. SCA-HU^+^ individuals presented significant increases in mean corpuscular volume (MCV) and mean corpuscular hemoglobin (MCH) and significant decreases in leukocyte, eosinophil, basophil, atypical lymphocyte, lymphocyte, and monocyte counts compared to SCA-HU^−^ individuals. Biochemical marker analysis showed lower concentrations of aspartate transaminase (AST) and lactate dehydrogenase (LDH) in the SCA-HU^+^ group than in the SCA-HU^−^ group (*p* < 0.05). In addition, significant increase was observed in creatinine levels of SCA-HU^+^ individuals compared to SCA-HU^−^ individuals (Table 2).

### 3.2. CML Levels in Individuals with HbSS and HbAA and Its Association with Laboratory Markers

**Figure 1:**
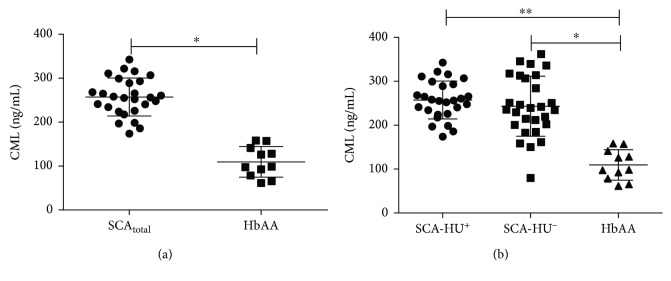
CML levels in individuals with SCA and HbAA. (a) Comparison of CML levels between SCA_total_ and HbAA groups shows higher levels of CML in individuals with SCA compared to individuals with HbAA. (b) Comparison of CML levels between SCA-HU^+^, SCA-HU^−^, and HbAA groups shows higher levels of CML in both individuals with SCA-HU^+^ and SCA-HU^−^ compared to individuals with HbAA.

**Figure 2:**
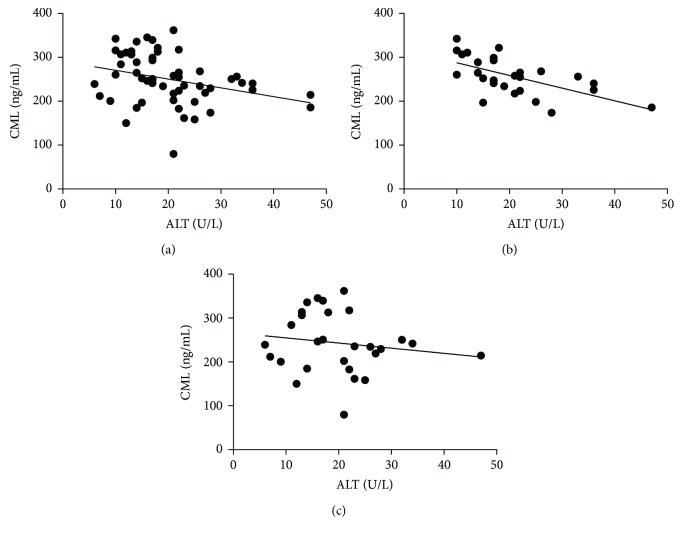
Correlation between CML levels and ALT in individuals with (a) SCA_total_, (b) SCA-HU^+^, and (c) SCA-HU^−^. A negative correlation was found between CML and ALT in individuals with (a) SCA_total_ (*r* = −0.35; *p* = 0.0092), (b) SCA-HU^+^ (*r* = −0.61; *p* = 0.0007), and (c) SCA-HU^−^ (*r* = −0.14; *p* = 0.4790). CML: N^*ε*^-carboxymethyllysine; ALT: alanine transaminase.

Comparison of serum levels of CML among the different groups showed that CML levels are significantly higher in SCA_total_ compared to individuals with HbAA (Figure 1(a)). In addition, both SCA-HU^+^ and SCA-HU^−^ presented higher levels of CML compared to individuals with HbAA (Figure 1(b)). Correlation analyses between CML and laboratory parameters showed a negative correlation between CML levels and ALT in SCA_total_ (*r* = −0.35; *p* = 0.0092), SCA-HU^+^ (*r* = −0.61; *p* = 0.0007), and SCA-HU^−^ (*r* = −0.14; *p* = 0.4790) groups (Figure 2(a)–2(c), respectively).

### 3.3. Association of CML Levels with *β*^S^ haplotypes

Tables 3(a), 3(b), and 3(c) show the distribution of genotypes of *β*^S^ haplotypes in SCA_total_, SCA-HU^+^, and SCA-HU^−^, respectively, according to CML concentrations. The results showed a significant association between *β*^S^ haplotypes and CML concentration (*χ*^2^ = 7.909, *p* = 0.048) in the SCA_total_ group (Table 3(a)). The Bantu/Benin genotype showed the highest association with CML.

## 4. Discussion

The present study sought to investigate the effect of CML on laboratory parameters in individuals with SCA according to HU use and *β*^S^ haplotypes. Laboratory marker analysis showed significant increase in MCV and MCH in the SCA-HU^+^ group compared to the SCA-HU^−^ group. This corroborates the report of Santos and Maia [33] which found significant change in MCV and MCH in individuals with SCA under HU therapy. HbF has been recognized as among the most important known modifiers of the clinical course of SCD. However, the HbF level did not significantly increase in SCA-HU^+^ in our research which is in agreement with the report of Steinberg et al. [34] in which the increase in HbF was not significant. This could be attributed to nonadherence to the daily dosage [35], variation in the length of HU treatment [20], lack of good bone marrow reserve, and/or patients on maximum tolerated dose [36]. However, various studies reported an increase in HbF levels after HU use [37].

WBC, neutrophils, monocytes, and eosinophils are known to activate the endothelial cells [20]. In the present study, WBC, eosinophil, basophil, monocyte, atypical, and typical lymphocyte counts were significantly reduced in patients receiving HU suggesting a reduction in vasoocclusive crisis [38, 39]. Our results corroborate the findings of Davies and Gilmore [36] and Silva-Pinto et al. [40]. Zimmerman et al. [41] suggested that neutrophils adhere to the vascular endothelium thereby potentially impairing the smooth flow of the sickle cells. Neutrophil adherence to the vascular endothelium has also been reported to cause increase in whole blood viscosity and release of cytokines that are known to be involved in inflammatory response including pain pathways [42].

Creatinine is the most commonly used endogenous marker to assess renal function. A reduction in the glomerular filtration rate (GFR) is usually associated with elevation in the serum creatinine level [43]. However, in SCD, renal failure is usually preceded by subclinical glomerular hyperfiltration [44]. Therefore, the elevated creatinine level, as we observed in SCA-HU^+^, is not always representative of a true reduction in GFR due to decreased tubular secretion caused by hyperfiltration [44]. Drugs such as trimethoprim, cimetidine, and other H_2_ blockers have been reported to inhibit tubular secretion of creatinine thereby causing an increase in the serum creatinine level [45, 46], and this could also be the case of HU. Furthermore, LDH and AST levels were significantly reduced in SCA-HU^+^ compared to SCA-HU^−^, and these findings are in agreement with the results of other works that reported improvement in hepatic markers such as LDH and AST [47, 48]. Hence, the results suggest that HU reduces liver damage and hemolysis since they are both equally markers of hemolysis.

Reports have already shown that a high level of circulating CML is involved in the pathogenesis of several age-enhanced diseases such as diabetic nephropathy, atherosclerosis, diabetic retinopathy, hemodialysis-associated amyloidosis, chronic renal failure, and Alzheimer's disease [49–53]. In our study, the CML, a biomarker of oxidative stress, was significantly higher in both SCA-HU^+^ and SCA-HU^−^ compared to the individuals with HbAA. This finding is in agreement with previous reports that discovered significant increase in CML in individuals with SCA compared to individuals with HbAA [15, 19] and suggests that CML may also have a role in the chronic vascular dysfunction observed in SCA [19]. In addition, studies reported that CML has a crucial role in the etiology of chronic microvascular complications in diabetes and other diseases [51, 52]. Our results further revealed that there is no significant difference between CML in the SCA-HU^+^ and SCA-HU^−^ groups. Nur et al. [15] also reported no significant difference between the SCA-HU^+^ and SCA-HU^−^ groups. This result suggests that HU has no effect on CML levels in individuals with SCA. The absence of significant difference could be due to low adherence to the daily HU dosage in some children, variation in the length of HU treatment [20, 35], or because CML are stable compounds compared to ROS, in addition to the fact that CML can be formed by either AGE or an advanced lipoxidation end product (ALE) synthesis pathway [14].

Correlation analysis showed significant negative correlation between CML and ALT in the SCA-HU^+^ and SCA_total_ groups. Since CML is a marker of oxidative stress that contributes to the initiation and progression of liver injury [54] and ALT is a liver biomarker [55], we had expected a positive relationship between CML and ALT. However, an experimental study on mice observed an association between regular AGE diet and higher ALT levels [56]. This tends to concur with our observation of an inverse correlation between CML and ALT. However, further investigations are needed to elucidate this association.

Association analysis showed a significant association between CML and *β*^S^ haplotypes in SCA_total_. Subjects with the Bantu/Benin haplotype were most associated with CML higher than 239.33 ng/mL. This can be explained by the fact that the Bantu haplotype is associated with the most severe clinical profile and consequently to higher inflammation and oxidative processes [57].

## 5. Conclusion

In summary, CML has a role to play in SCA complications that seems not to be influenced by HU treatment. Furthermore, our report suggests an association between CML and *β*^S^ haplotypes.

## Figures and Tables

**Table 1:** Hematological characteristics of individuals with SCA with or without HU treatment.

Hematological variables	SCA-HU^+^ *N* = 27, *M* (25^th^–75^th^ per)	SCA-HU^−^ *N* = 28, *M* (25^th^–75^th^ per)	*p* value^∗^
Red blood cell (×10^12^/L)	2.69 (2.15–3.18)	2.66 (2.45–3.22)	0.533
Hemoglobin (g/dL)	8.7 (8–10.4)	8.6 (7.9–9.3)	0.337
Hematocrit (%)	24.9 (22.6–30.4)	24.5 (22.2–27.9)	0.522
MCV (fL)	100.9 (89.7–106.7)	92.2 (89.00–94.50)	**0.003**
MCH (*ρ*g)	34.3 (30.9–37.1)	31.6 (29.7–32.9)	**0.002**
MCHC (g/dL)	34 (33.5–35.4)	34.1 (33.3–35.6)	0.890
RDW	20.6 (17.3–22.8)	21.1 (18.70–23.3)	0.398
Reticulocytes (%)	7.1 (5.7–8.7)	8.00 (5.4–9.6)	0.728
WBC (×10^6^/L)	9335 (5860–13100)	12300 (10500–14500)	**0.014**
Neutrophils (×10^6^/L)	4400 (2602–6514)	5280 (4170–6380)	0.250
Eosinophils (×10^6^/L)	306 (96–693)	679 (272–1408)	**0.017**
Basophils (×10^6^/L)	32 (0–96)	120 (0–158)	**0.041**
Atypical lymphocytes (×10^6^/L)	0 (0–54)	104 (0–380)	**0.014**
Typical lymphocytes (×10^6^/L)	3930 (2533–5328)	4921 (3770–6950)	**0.033**
Monocytes (×10^6^/L)	720 (393–879)	896 (582–1390)	**0.041**
Platelets (×10^3^/mL)	374 (275–441)	392 (321–463)	0.610
MPV	5.8 (5.2–7)	5.70 (5.20–6.90)	0.856
HbS (%)	80.3 (73.2–86.95)	85.3 (80.1–88.2)	0.286
HbF (%)	9.6 (6.8–15.9)	8.2 (5.7–9.9)	0.072
HbA_2_ (%)	4.4 (3.9–5.4)	5.55 (4.15–6.90)	0.078

SCA-HU^+^: individuals with SCA with HU treatment; SCA-HU^−^: individuals with SCA without HU treatment; MCV: mean corpuscular volume; MCH: mean corpuscular hemoglobin; MCHC: mean corpuscular hemoglobin concentration; RDW: red blood cell distribution width; WBC: white blood cell; MPV: mean platelet volume; HbS: hemoglobin variant S; HbF: fetal hemoglobin; HbA_2_: normal hemoglobin A_2_; *N*: number; *M*: median; per: percentile. ^∗^Mann-Whitney *U* test.

**Table 2:** Biochemical characteristics of individuals with SCA with or without HU treatment.

Biochemical variables	SCA-HU^+^ *N* = 27, *M* (25^th^–75^th^ per)	SCA-HU^−^ *N* = 28, *M* (25^th^–75^th^ per)	*p* value^∗^
Glucose (fasting) (mg/dL)	78 (72–85)	71 (63–85)	0.089
Total cholesterol (mg/dL)	129 (112–138)	127.5 (114.5–144)	0.637
HDL-C (mg/dL)	32 (26–45)	32.5 (28–42)	0.827
LDL-C (mg/dL)	71.6 (57.8–91.4)	74.2 (63.1–92.5)	0.479
VLDL-C (mg/dL)	14.2 (11.8–21.2)	16.6 (12.8–20)	0.381
Triglyceride (mg/dL)	71 (59–106)	83 (64–100)	0.381
ALT (U/L)	18 (14–25)	19.5 (41–57)	0.873
AST (U/L)	38 (30–48)	51.5 (41–57)	**0.001**
Serum iron (*μ*g/dL)	99.6 (75.3–134.3)	74.15 (52.15–114.15)	0.065
Ferritin (ng/mL)	265.75 (126.9–571.35)	160.25 (122–266.65)	0.102
Total bilirubin (mg/dL)	1.9 (1.23–3.7)	2.32 (1.53–3.67)	0.350
Direct bilirubin (mg/dL)	0.45 (0.32–0.59)	0.45 (0.40–0.52)	0.730
Indirect bilirubin (mg/dL)	1.29 (0.78–3.1)	1.8 (1.07–3.27)	0.239
Total protein (g/dL)	7.47 (6.89–8)	7.64 (7.03–7.83)	0.849
Albumin (g/dL)	4.4 (4–4.4)	4.4 (4.1–4.50)	0.582
Globulin (g/dL)	3.3 (2.6–3.8)	3.25 (2.5–3.55)	0.735
Urea (mg/dL)	19 (14–24)	17 (15–21)	0.468
Creatinine (mg/dL)	0.47 (0.41–0.56)	0.39 (0.34–0.48)	**0.004**
C-reactive protein (mg/L)	4.07 (2.69–8.3)	3.62 (2.07–5.89)	0.246
LDH (U/L)	853 (715–1128)	1270 (1091–1612)	**≤0.001**
A/G ratio	1.3 (1.1–1.7)	1.30 (1.15–1.70)	0.754
AAT (mg/dL)	138.5 (119.5–159.5)	154.5 (118–174)	0.331
Nitric oxide (*μ*M)	20.44 (15.85–22.72)	19.02 (14.11–25.29)	0.755

SCA-HU^+^: individuals with SCA with HU treatment; SCA-HU^−^: individuals with SCA without HU treatment; HDL-C: high-density lipoprotein-cholesterol; LDL-C: low-density lipoprotein-cholesterol; VLDL-C: very low-density lipoprotein-cholesterol; ALT: alanine transaminase; AST: aspartate transaminase; LDH: lactate dehydrogenase; A/G ratio: albumin/globulin ratio; AAT: alpha 1-antitrypsin; *N*: number; *M*: median; per: percentile. ^∗^Mann-Whitney *U* test.

**Table:** (a) SCA_total_

Genotype	CML		Total, *n* (%)	*χ* ^2^	*p*
	≤239.33 ng/mL *n* (%)	>239.33 ng/mL *n* (%)			
Bantu/Bantu	3 (5.8)	8 (15.4)	11 (21.2)	7.909	0.048^∗∗^
Bantu/Benin	5 (9.6)	16 (30.8)	21 (40.4)		
Bantu/atypical	2 (3.8)	0 (0.0)	2 (3.8)		
Benin/Benin	10 (19.2)	8 (15.4)	18 (34.6)		
Total	20 (38.5)	32 (61.5)	52 (100)		

CML: N^*ε*^-carboxymethyllysine; *χ*^2^: Pearson chi-square. ^∗∗^Bantu/Bantu versus Bantu/Benin, Bantu/atypical, and Benin/Benin.

**Table:** (b) SCA-HU^+^

Genotype	CML		Total, *n* (%)	*χ* ^2^	*p*
	≤256.10 ng/mL *n* (%)	>256.10 ng/mL *n* (%)			
Bantu/Bantu	1 (4.2)	3 (12.5)	4 (16.7)	1.929	0.381^∗∗^
Bantu/Benin	4 (16.7)	5 (20.8)	9 (37.5)		
Benin/Benin	7 (29.2)	4 (16.7)	11 (45.8)		
Total	12 (50.0)	12 (50.0)	24 (100)		

CML: N^*ε*^-carboxymethyllysine; *χ*^2^: Pearson chi-square. ^∗∗^Bantu/Bantu versus Bantu/Benin, Bantu/atypical, and Benin/Benin.

**Table:** (c) SCA-HU^−^

Genotype	CML		Total, *n* (%)	*χ* ^2^	*p*
	≤237.39 ng/mL *n* (%)	>237.39 ng/mL *n* (%)			
Bantu/Bantu	3 (10.7)	4 (14.3)	7 (25.0)	2.619	0.454^∗∗^
Bantu/Benin	5 (17.9)	7 (25.0)	12 (42.9)		
Bantu/atypical	2 (7.1)	0 (0.0)	2 (7.1)		
Benin/Benin	4 (14.3)	3 (10.7)	7 (25.0)		
Total	14 (50.0)	14 (50.0)	28 (100)		

CML: N^*ε*^-carboxymethyllysine; *χ*^2^: Pearson chi-square. ^∗∗^Bantu/Bantu versus Bantu/Benin, Bantu/atypical, and Benin/Benin.

## Data Availability

The laboratory data used to support the findings of this study are available from the corresponding author upon request.
